# The effect of MEP pathway and other inhibitors on the intracellular localization of a plasma membrane-targeted, isoprenylable GFP reporter protein in tobacco BY-2 cells

**DOI:** 10.12688/f1000research.2-170.v2

**Published:** 2013-11-15

**Authors:** Michael Hartmann, Andrea Hemmerlin, Elisabet Gas-Pascual, Esther Gerber, Denis Tritsch, Michel Rohmer, Thomas J Bach

**Affiliations:** 1 Département Réseaux Métaboliques, Institut de Biologie Moléculaire des Plantes, CNRS UPR 2357, Université de Strasbourg, F-67083 Strasbourg, France; 2 Current address: Institute of Biological Chemistry, Washington State University, Pullman WA, 99164-6340, USA; 3 Current address: Department of Horticulture and Crop Science, Ohio State University, Wooster OH, 44691, USA; 4 Current address: Deinove SA, F-34830 Clapiers, France; 5 UMR 7177 CNRS, Laboratoire de Chimie et Biochimie des Microorganismes, Institut de Chimie de Strasbourg, Université de Strasbourg, F-67008 Strasbourg, France

## Abstract

We have established an *in vivo* visualization system for the geranylgeranylation of proteins in a stably transformed tobacco BY-2 cell line, based on the expression of a dexamethasone-inducible GFP fused to the carboxy-terminal basic domain of the rice calmodulin CaM61, which naturally bears a CaaL geranylgeranylation motif (GFP-BD-CVIL). By using pathway-specific inhibitors it was demonstrated that inhibition of the methylerythritol phosphate (MEP) pathway with known inhibitors like oxoclomazone and fosmidomycin, as well as inhibition of the protein geranylgeranyltransferase type 1 (PGGT-1), shifted the localization of the GFP-BD-CVIL protein from the membrane to the nucleus. In contrast, the inhibition of the mevalonate (MVA) pathway with mevinolin did not affect the localization. During the present work, this test system has been used to examine the effect of newly designed inhibitors of the MEP pathway and inhibitors of sterol biosynthesis such as squalestatin, terbinafine and Ro48-8071. In addition, we also studied the impact of different post-prenylation inhibitors or those suspected to affect the transport of proteins to the plasma membrane on the localization of the geranylgeranylable fusion protein GFP-BD-CVIL.

## Introduction

In higher plants, the synthesis of the general isoprenoid precursors isopentenyl diphosphate (IPP) and dimethylallyl diphosphate (DMAPP) is accomplished through two different routes, the cytosolic mevalonic acid (MVA) pathway and the plastidial methylerythritol phosphate (MEP) pathway (cf. ^1^).

The MVA pathway supplies the biosynthetic precursors for isoprenoids in the majority of eukaryotes (including all animals, the archea, some eubacteria, fungi and the cytosol/mitochondria of some algae and higher plants). It can be found in several important human parasites, such as *Trypanosoma* and *Leishmania* ^2^. In humans, the MVA pathway operates alone and produces a variety of critical end products, including cholesterol, steroid hormones, dolichols and the prenyl moiety of cancer-associated cell signaling proteins like RAS ^3– 6^. In plants, the cytosolic IPP provided by the MVA pathway serves as precursor for the synthesis of sterols, brassinosteroids, polyprenols, dolichols and most sesquiterpenes and to some extent as a substrate for protein prenylation (cf. ^7, 8^). Moreover, cytosolic IPP is imported into the mitochondria, where it serves as precursor for ubiquinone ^9– 11^.

The alternative pathway (or MEP-pathway) for the synthesis of isoprenoids occurs in eubacteria, cyanobacteria, and the plastids of phototrophic algae and plants ^12– 19^. In plants, the precursors, provided by the plastidial MEP pathway are used for the biosynthesis of essential isoprenoids of the photosynthetic apparatus such as carotenoids, the side-chains of chlorophyll and plastoquinone, as well as for isoprene, tocopherols, phylloquinones and the phytohormones ABA and gibberellin ^20^, with ABA being a cleavage product of carotenoids, like the more recently discovered strigolactones ^21, 22^. In addition to these ubiquitous compounds in plants, the MEP pathway is the route for the biosynthesis of the vast majority of plant terpenoids, including countless secondary metabolites with defensive, allelopathic or signaling properties ^11, 18, 23^ (and Hemmerlin *et al.* ^24^ for review of literature). Furthermore, precursors derived from the MEP pathway are used for the post-translational modification of certain proteins by the covalent addition of a farnesyl-(C _15_) or geranylgeranyl (C _20_) residue, a process commonly referred to as protein prenylation (cf. the accompanying paper ^25^ and literature cited therein).

In preceding studies investigating the differential effect of a series of inhibitors of isoprenoid biosynthesis and function in cell cycle progression in unsynchronized and synchronized BY-2 cells ^26^, it had been observed that blockage of protein farnesylation by chaetomellic acid not only led to a considerable percentage increase in dead cells during the culture period, but also to a specific arrest in the transition from G2 into M phase. By contrast, mevinolin for instance (which inhibits the key-regulatory enzyme HMG-CoA reductase in the cytosolic MVA pathway) arrested cells mainly at the transition from G1 to S phase ^27^. This latter finding is most likely due to the lack of some MVA-derived signal formed at the end of mitotic phase that might be implied in the regulation of the cytoplasmic pH ^28^. These and other observations prompted a series of further studies in which the possibility that the plastidial MEP pathway could complement MVA deficiency was tested. Indeed it was demonstrated that exogenously added deoxyxylulose (DX, the dephosphorylated first product of the MEP pathway) could overcome mevinolin-induced growth inhibition, even more efficiently than exogenous MVA ^29^. To enter the plastidial MEP route, this DX needs conversion into its phosphate (DXP) by a cytosolic xylulose kinase ^30^, followed by translocation into plastids. As a logical follow-up, and in view of early reports that in plants geranylgeranylated proteins seem to be present in higher quantities than farnesylated ones ^31, 32^, we embarked on studying this phenomenon more closely. Our interest is not only focused on elucidation of the biosynthetic origin of farnesyl and geranylgeranyl residues, but also on the action of inhibitors on precursor availability and on the specificity of prenyltransferases. For instance, we could demonstrate the incorporation of ^14^C-DX into proteins in BY-2 cells ^29^.

The central element of our recent studies was an *in vivo*-visualization system based on a stably transformed tobacco BY-2 (TBY-2) cell line for monitoring the prenylation status of a GFP fusion protein. Isoprenylation of proteins, which occurs in all eukaryotic cells, involves the covalent attachment of a C _15_ (farnesyl) or C _20_ (geranylgeranyl) group to a C-terminal CaaX motif, followed by a series of post-prenylation reactions. This lipidic post-translational modification plays an important role in the correct membrane targeting of certain proteins and in their interactions with other proteins.

This system consisted of a dexamethasone-inducible cell line that expressed a reporter protein (GFP) fused to the carboxy-terminal basic domain of the rice calmodulin (CaM61), which naturally bears a CaaL geranylgeranylation motif (GFP-BD-CVIL). After induction, the prenylated fusion protein predominantly associated with the plasma membrane. By using pathway-specific inhibitors, we demonstrated that inhibition of the MEP pathway with oxoclomazone and fosmidomycin, as well as inhibition of the protein geranylgeranyltransferase type 1 (PGGT-1), triggered a shift in the localization of the GFP-BD-CVIL protein from the plasma membrane to the nucleus ^1^. By contrast, inhibition of the MVA pathway (by mevinolin) or protein farnesyltransferases did not affect the localization of the chimeric fusion protein. Among other experiments, complementation assays with pathway-specific intermediates were performed and clearly indicated that the precursors for the geranylgeranylation of the fusion protein in tobacco BY-2 cells were predominantly provided by the MEP pathway ^1^.

However, at the end of this previous study several questions remained unsolved that will be addressed in more detail in the present work, including the impact of inhibitors of sterol biosynthesis and post-prenylation reactions on the subcellular localization of the His _6_-tagged GFP-BD-CVIL fusion protein. In order to prove that the present bioassay was not only able to serve as a qualitative approach for the identification of new drug candidates, but also as a statistical tool to compare the potential drug candidates with other known inhibitors *in vivo*, we also performed a quantitative analysis of the intracellular distribution of GFP-DB-CVIL in response to different concentrations of novel prodrugs, targeting the early steps of the MEP pathway.

## Results

### The robustness of the BY-2 cell test system - effects of treatments with sterol biosynthesis inhibitors

By blocking the early steps of sterol biosynthesis with pathway-specific inhibitors we tried to determine the impact of sterol depletion in the plasma membrane as well as of the modulation of the pool of endogenously available FPP on the localization of the H _6_-GFP-BD-CVIL fusion protein.

First of all, squalestatin-1 (SQ), also referred to as zaragozic acid ^33^, was used to specifically inhibit the conversion of farnesyl diphosphate (FPP) to squalene by the first committed enzyme in the sterol/triterpene pathway, squalene synthase (SQS). SQ is a competitive inhibitor of SQS and structurally mimics the reaction intermediate presqualene diphosphate ^33– 37^.

Seven-day-old BY-2 cells were diluted at a ratio of 1 to 6 in fresh medium and treated with 1µM SQ for 18–24 h (in different independent experiments). 15 h before observation of the cells by fluorescence microscopy, expression of the prenylable fusion protein H _6_-GFP-BD-CVIL was induced by addition of 10µM dexamethasone (150 rpm, 26°C, growth in the dark).

**Figure 1.**
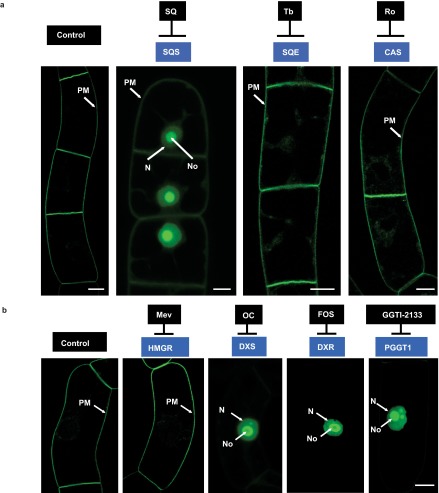
a) Localization of H _6_-GFP-BD-CVIL in transgenic BY-2 cells after treatment with inhibitors of sterol biosynthesis. Control: GFP fluorescence is almost exclusively associated with the plasma membrane (PM). SQ: Cells treated with squalestatin-1 (SQ), a specific inhibitor of squalene synthase (SQS) show partial translocation of GFP fluorescence to the nucleus (N) and the nucleolus (No). Tb: Cells treated with terbinafine (Tb), an inhibitor of squalene epoxidase (SQE), showing GFP fluorescence associated with the plasma membrane (very faint fluorescence is also seen in the cytoplasm). Ro: Localization of H _6_-GFP-BD-CVIL in cells treated with Ro48-8071, a general inhibitor of oxidosqualene cyclases (OSC), such as cycloartenol synthase (CAS) in plants, showing GFP fluorescence associated with the plasma membrane. **b) Localization of H _6_-GFP-BD-CVIL fusion protein in transgenic BY-2 cells after treatment with inhibitors of key enzymes of the MVA and MEP pathways.** Control cells were induced 15 h before observation by confocal microscopy as described. Treatment with inhibitors occurred 3 h prior to dexamethasone induction ^116^. Mevinolin (Mev, 5µM) did not cause any detectable effect on the localization of GFP-BD-CVIL fusion protein when compared to the untreated control. GFP fluorescence was predominantly located at the periphery of the cells. By contrast, Fosmidomycin (Fos) as well as oxoclomazone (OC) (here both at 30µM) caused a mislocalization of the fusion protein to the nucleus and in particular to the nucleolus. The same phenotype could also be observed after application of 40µM GGTI-2133, a peptidomimetic inhibitor of protein geranylgeranyl transferase 1 (PGGT1). White bar = 10µm.

The results of this treatment are shown in Figure 1a. Two general observations can be made. First of all, treatment with SQ resulted in a partial mislocalization of the prenylated fusion protein from the PM to the nuclear compartment, with the strongest signal being emitted by the nucleolus. In addition, the overall morphology of the treated cells was changed. Cells treated with 0.5 or 1µM SQ showed stunted growth with clearly reduced length-to-diameter ratios as compared to control cells. This latter effect has already been described ^38^.

In order to examine whether this effect was due to sterol depletion or a side effect of squalestatin on prenyl transferases in general (cf. ^39^), BY-2 cells were treated with terbinafine (Tb), a specific, non-competitive inhibitor of the fungal squalene epoxidase (SQE). In mammals, however, it acts as a competitive inhibitor of this enzyme ^40^. Its inhibitory efficiency in plant cells has been demonstrated with celery ( *Apium graveolens*) cell suspension cultures ^41^, wheat ( *Triticum aestivum*) seedlings ^42^, cat’s claw ( *Uncaria tomentosa*) ^43^ and with our model system, BY-2 cell suspension cultures ^38^. Treatments with Tb were performed at 30µM up to 24 h (as well as 18 h). The cells treated with Tb showed a very slightly stunted growth as compared to the control cells and displayed faint GFP signals in the cytosol, close to the PM. However, no mislocalization of the prenylable fusion protein to the nuclear compartment could be observed under the chosen experimental conditions ( Figure 1a).

Finally, Ro48-8071 (Ro) was used to inhibit one of the key steps of sterol biosynthesis, the conversion of the linear oxidosqualene into cycloartenol, the first cyclic precursor of phytosterols. This reaction is catalyzed by cycloartenol-synthase (CAS) in plants ^44^. Ro is a potent inhibitor of oxidosqualene cyclases (OSC) in general, including lanosterol synthase (LAS) in mammals and fungi ^45^. The structure of Ro48-8071, which is an orally active inhibitor of human hepatic OSC, has been determined in complex with the squalene-hopene cyclase (SHC), the prokaryotic counterpart of OSCs, responsible for the conversion of squalene into cyclic compounds in bacteria, and it is suggested that Ro reacts with the expected binding site for squalene ^46^. Ro treatments were performed at 2µg/ml under the same experimental conditions as described previously for SQ and Tb applications. Cells treated with Ro for 18/24 h looked morphologically more or less like the control cells. The GFP fusion protein was mainly localized at the level of the plasma membrane, although there were also faint signals (small speckles) of fluorescence near the PM like those observed for the previous Tb treatments. However, no mislocalization of the GFP fluorescence to the nucleus/nucleolus was observed ( Figure 1a). When compared to treatments like those described by Gerber *et al*. ^1^ and repeated here ( Figure 1b), the effects were slightly less pronounced.

**Figure 2.**
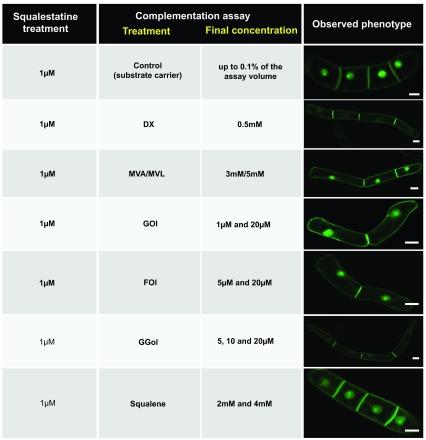
Chemical complementation of squalestatin-induced H _6_-GFP-BD-CVIL mislocalization. Squalestatin was added at 1µM throughout the series of experiments. Partial delocalization (from the PM to the nucleus/nucleolus) with pathway intermediates, isoprenols and squalene in an attempt to chemically complement the effect of squalestatin. MVA: mevalonic acid; MVL: mevalonolactone; DX: 1-deoxyxylulose; Gol: geraniol; Fol: farnesol; GGol: geranylgeraniol. White bars = 20µm.

Various complementation experiments were performed with SQ-treated cells. Whereas DX (0.5mM) and geranylgeraniol (GGol, 20µM) were able to re-establish the membrane localization in 100% of the cells (better than the control), neither geraniol (Gol, 20µM) nor mevalonolactone (MVL, 5mM) and its open carboxylic form mevalonate (MVA, 3mM) could complement the mislocalization under the chosen experimental conditions. Finally, squalene was added at 2–4mM, but did not overcome the SQ-induced effect either ( Figure 2).

### Post-prenylation inhibitors and transport of GFP-BD-CVIL to the plasma membrane

As mentioned earlier, the purification and analysis of the His _6_-tagged GFP-BD-CVIL fusion protein revealed that it was geranylgeranylated and carboxyl-methylated in BY-2 cells ^1^. In the past the study of post-prenylation inhibition has become a very attractive topic, as both reactions are essential for the localization of many prenylated proteins by mediating their attachment to membranes and protein-protein interactions.

**Figure 3.**
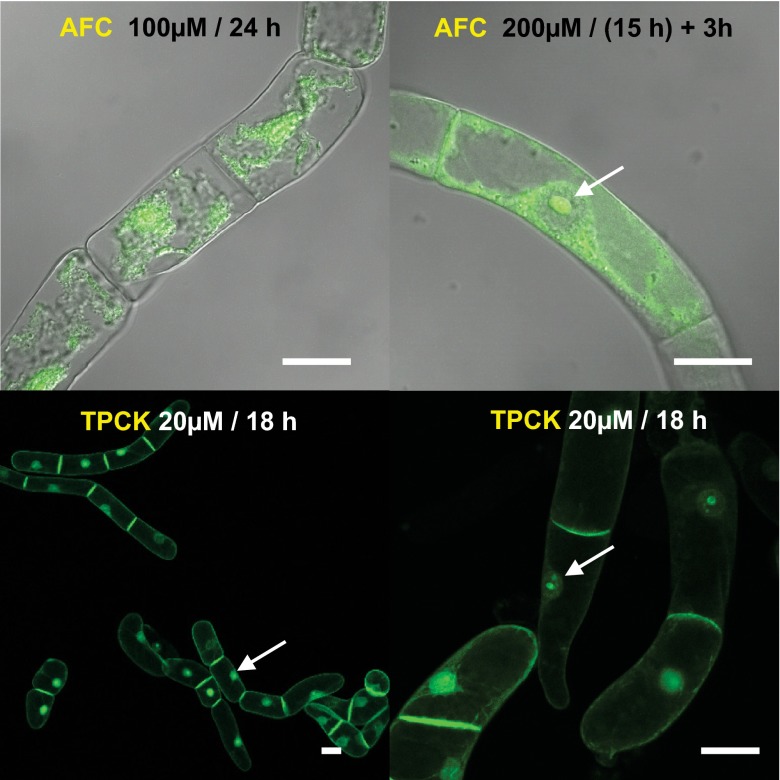
Effect of post-prenylation inhibitors on the localization of the geranylgeranylated fusion protein H _6_-GFP-BD-CVIL. AFC: *N*-acetyl- *S*-farnesyl- L-cysteine, an inhibitor of isoprenylcysteine carboxyl methyltransferase; TPCK: *N*-tosyl- L-phenylalanine chloromethyl ketone, a serine and cysteine proteinase inhibitor. White bars = 20µm.

In preliminary experiments we tested inhibitors of RAS converting enzyme 1 (RCE1), and of isoprenylcysteine carboxyl methyltransferase (ICMT), but most of them induced cell death when used in commonly cited concentrations and time-scales in our model system. This is most likely due to the efficient uptake and high metabolic activity of BY-2 cells ^29^ and requires adjustment of the experimental conditions. Nonetheless, the results obtained after various treatments indicated that both inhibition of RCE1 and ICMT affected the localization of H _6_-GFP-BD-CVIL ( Figure 3). For instance, short-term treatment (15 h induction, then treatment for 3 h with 200µM of the prenylcysteine analog *N*-acetyl- *S*-farnesyl- L-cysteine (AFC)) strongly changed the distribution pattern of the H _6_-GFP-BD-CVIL and besides the PM, both the cytosol as well as the nucleolus displayed strong GFP signals ( Figure 3). Interestingly, a similar effect on the localization of the prenylated GFP-CaM53 fusion protein of *Petunia* was observed by Rodríguez-Concepcíon *et al.* ^47^ in response to AFC treatment (200µM), using bombarded *Petunia* leaves. Long-term treatments of BY-2 cells (> 10 h) however led to loss of GFP fluorescence and cell death.

Inhibition of the –AAX-proteolysis by *N*-tosyl- L-phenylalanine chloromethyl ketone (TPCK ^48^) led to a partial mislocalization of the fusion protein to the nuclear compartment. However, optimal conditions to observe the induced effects still need to be identified.

### DXS as a molecular target for oxoclomazone and pyruvate derivatives

DXS (1-deoxy- D-xylulose 5-phosphate synthase) catalyzes the first step of the alternative MEP pathway, the condensation of glyceraldehyde 3-phosphate (GAP) and pyruvate, yielding 1-deoxy- D-xylulose 5-phosphate (DXP). DXS enzymes are highly conserved in bacteria and plants and analyses of their sequences revealed a weak homology with other thiamine-dependent enzymes, such as transketolases, and the pyruvate dehydrogenase E1 subunit ^49– 52^. These enzymes all catalyze similar biochemical reactions ^53, 54^ by using thiamine diphosphate (TPP) as a cofactor and pyruvate as a substrate. In addition to TPP, DXS also requires a divalent cation (Mg ^2+^ or Mn ^2+^) for optimum enzyme activity ^55, 56^. More recently, efforts were successful to partially crystallize DXS from *E. coli* and *Deinococcus radiodurans* (in complex with TPP), after *in situ* proteolysis of the purified enzyme by a fungal protease ^57, 58^, which led to a better understanding of the catalytic mechanism of DXS and possibly paves the way for the design of novel active inhibitors. As to date, only two inhibitors of this enzyme are known: oxoclomazone (OC), for the plant DXS ^59^ and fluoropyruvate, for the DXS of *E. coli* ^60^. OC, sometimes also referred to as 5-ketoclomazone, was only recently reported to have exhibited antibacterial activity against a pathogenic bacterium, *Haemophilus influenza* ^61^.

**Figure 4.**
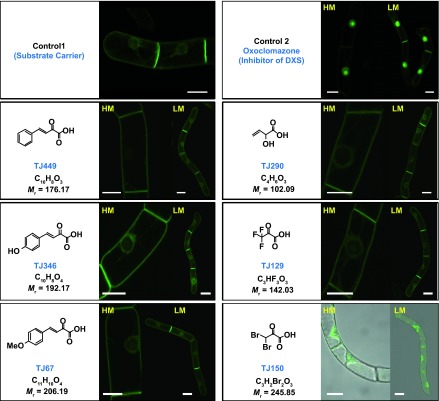
Confocal microscopy images showing the subcellular localization of the H _6_-GFP-DB-CVIL fusion protein, after treatment with different analogs of pyruvate based on known inhibitors of pyruvate decarboxylase and pyruvate dehydrogenase. The molecules (at a final concentration of 100µM) were dissolved and tested under standard conditions (3 h treatment, followed by induction with 10µM dexamethasone and examination by fluorescence microscopy 15 h later). Cells were also treated by 50µM oxoclomazone as described previously (positive control). None of the six tested compounds showed a mislocalization of the GFP fluorescence to the nucleus. The compound TJ150, however, appeared to be cytotoxic at the given concentration as cells displayed typical signs of cell death (over 90% of treated cells did not show any fluorescence).

The synthesis of different classes of pyruvate analogs was inspired by known inhibitors of pyruvate decarboxylases and pyruvate dehydrogenases (cf. ^62– 64^). These compounds were then tested with our bioassay for possible effects on the localization of the H _6_-GFP-BD-CVIL ( Figure 4), thus indicating an inhibitory effect on the biosynthesis of GGPP via the MEP pathway. All compounds (cf. Figure S4) were dissolved in their respective solvent (water or acetonitrile). The only exception was *p*-hydroxyphenylpyruvate, which did not dissolve but gave a homogenous, yellow suspension.

The cells were treated for 18 h, in the presence of 100µM of pyruvate analogs, and the next day examined by confocal fluorescence microscopy. None of the tested compounds induced a mislocalization of green fluorescence, as it was observed for the positive control, which was treated with 50µM OC. However, the dibromopyruvate-treated cell culture showed significant cytotoxic effects as over 90% of cells did not display any GFP-related fluorescence, indicative of cell death. The fluorescence in the remaining cells appeared in cytoplasmic strands and in the cytoplasm surrounding the nucleus ( Figure 4).

### DXR as a molecular target for fosmidomycin and derived compounds

1-Deoxy- D-xylulose 5-phosphate reductoisomerase (DXR) is the second enzyme of the MEP pathway and catalyzes the conversion of 1-deoxy- D-xylulose 5-phosphate (DXP) to 2- *C*-methyl- D-erythritol 4-phosphate (MEP). Part of this transformation is an intramolecular rearrangement, yielding 2- *C*-methyl- D-erythrose 4-phosphate, which is then reduced to MEP in an NADPH-dependent reaction step ^65– 68^. Several studies have revealed detailed information about the structure and biochemical properties of the DXR enzyme from *E. coli* ^67– 69^. Those results – including the three-dimensional structures of DXR in a ternary complex with DXP/fosmidomycin (Fos) and the co-factor NADPH – suggest a physiologically active homodimer, with each subunit consisting of three distinct domains ^70, 71^. In addition to NADPH, DXR requires a divalent metal cation for activity, such as Mn ^2+^, Mg ^2+^ or, *in vitro*, Co ^2+^, which is bound by three highly conserved amino acid residues ^70, 72^. This metal cation is chelated by DXP before the first step of the conversion to MEP – an intramolecular rearrangement – takes place. According to the structural data, binding of DXR to its substrate (or to Fos) involves a major conformational rearrangement of the enzyme in the presence of NADPH. Fos acts as a competitive inhibitor, chelating a bivalent cation with its hydroxamate group and binding slowly but very tightly to the catalytic site of DXR ^70^. The substrate binding site of the DXR enzyme can be divided into three distinct regions: first of all, a positively charged pocket which interacts with the phosphonate function of Fos (“phosphate-recognition site”), a hydrophobic region covering the backbone of the molecule, as well as an amphipathic region that binds the hydroxamic acid moiety of the molecule. This results in a conformation with a flexible loop covering the central catalytic site, thus forming a barrier with the surrounding solvent ^71^.

**Figure 5.**
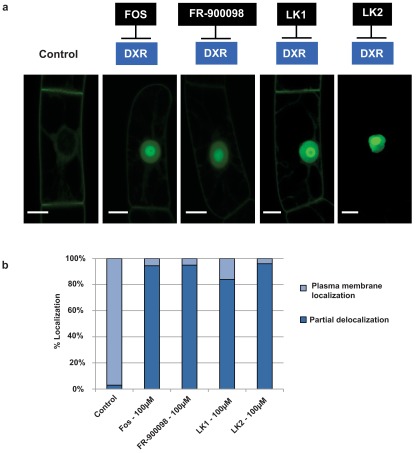
Localization of H _6_-GFP-BD-CVIL in transformed BY-2 cells after treatment with different inhibitors of DXR. **a**) Confocal images were taken after treatment with different inhibitors of DXR, under standard conditions (3 h pretreatment, followed by 15 h induction with 10µM dexamethasone). Control: Cells were treated with the same volume of solvent (DMSO). Inhibitors: All inhibitors (Fosmidomycin = FOS, FR-900098, LK1 and LK2) were tested at 100µM and caused a significant translocation of the GFP fusion protein to the nucleus/nucleolus. White bars = 10µm. **b**) Quantitative analysis of H _6_-GFP-BD-CVIL localization in BY-2 cells treated with inhibitors of DXR. Percentage of cells showing a partial localization of the fluorescence to the nucleus and nucleolus (without taking care of the total intensity of this translocation) and the plasma membrane. For each treatment more than 100 individual cells were counted.

Fos is known to inhibit the DXR enzyme from higher plants ^73, 74^, and experiments with various plant species have demonstrated its potential as herbicide, as approved by the chlorotic and bleaching phenotypes that have been observed after its application ^75^. In addition, Fos successfully inhibited the isoprenylation of the GFP-BD-CVIL fusion protein in our fluorescent bioassay at concentrations in the micromolar range ^1^. Fos and its methylated derivative FR-90098 are phosphonohydroxamic acids. In both compounds a hydroxamate function is linked to a phosphonic acid function by a propyl chain. Fos and FR-90098 as well as two hydroxamate derivatives 4-(hydroxyamino)-4-oxobutyl-phosphonic acid (LK1) and 4-[hydroxy(methyl)amino]-4-oxobutyl-phosphonic acid (LK2 ^76^) have been tested on the transgenic BY-2 cell line expressing the H _6_-GFP-BD-CVIL marker protein using the well-established standard conditions. At 100µM, the majority of fluorescence emitted by the GFP marker protein of the cells treated with Fos, with FR-900098 or LK2 accumulated in the nucleus, indicating an efficient inhibition of the MEP pathway ( Figure 5a,b). At this concentration, the mislocalization affected about 95% of the observed cells. The only exception was LK1, which needed considerably higher concentrations to cause a similar effect.

A major disadvantage of phosphonate drugs is that the phosphonate group is highly deprotonated at physiological pH. Because of the resulting high polarity (and/or low lipophilicity), the transport across biological membranes and the general bioavailability are restricted (cf. ^77^). A common strategy to overcome this problem is masking the phosphonic acid moiety by esterification. For instance, double ester drugs of FR-900098 have shown a 2 to 3 fold higher biological activity compared to their unmodified models in experiments with malaria-infected mice ^78^.

**Figure 6.**
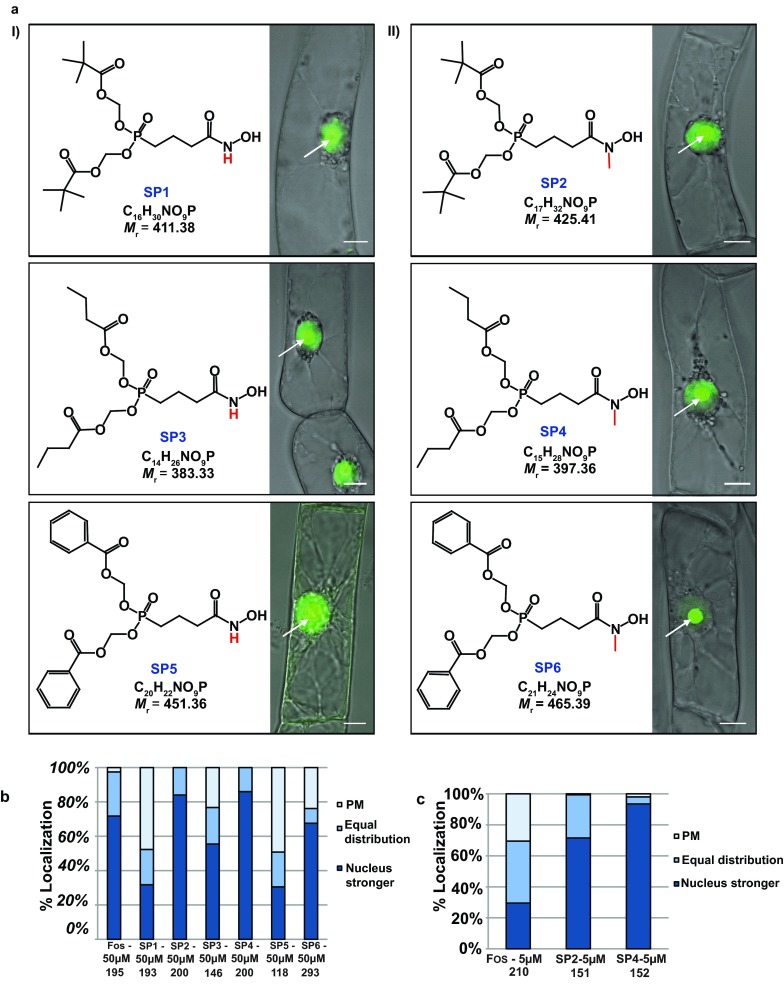
a) Confocal microscopy images showing the subcellular localization of H _6_-GFP-DB-CVIL fusion protein after treatment with a series of prodrug molecules (SP1–SP6) derived from Fos, an effective inhibitor of DXR. Six molecules have been tested in total. The tested molecules can be divided into two groups. **I)** Three non-methylesterprodrugs (derived from LK1: SP1, SP3 and SP5). **II)** Three molecules with a methyl group bound to the N-atom (derived from LK2: SP2, SP4 and SP6). The respective phosphonate groups were masked as acyloxymethyl phosphonate esters ^79^. Cells were pre-treated for 3 h with 50µM of each inhibitor (solvent: methanol) before expression of H _6_-GFP-DB-CVIL was induced by addition of dexamethasone (10µM final concentration). At the chosen concentration, the majority of all treated cells showed partial or very dominant delocalization of the fluorescence from the plasma membrane to the nucleus/nucleolus. The images are overlays from pictures in transmission light and fluorescence mode and were acquired as described in the main text. Bars = 10µm. **b) Statistical approach to compare the impact of six different pro-drugs on the *in vivo* localization of the prenylable fusion protein H _6_-GFP-BD-CVIL.** The cells (> 100) were treated with 50µM of the respective inhibitor. Cells were counted by view and sorted in three main categories (dominantly membrane localized (PM), nucleus localized and intermediate localization). The number of cells analyzed in each condition is indicated. **c) Statistical approach to compare the impact of six different pro-drugs on the *in vivo* localization of the prenylable fusion protein H _6_-GFP-BD-CVIL**. The cells were treated with 5µM of the respective inhibitor. Cells (> 100) were counted by view and sorted in three main categories as described above.

Several double ester prodrugs based on the original structures of LK1 (derived from fosmidomycin) and LK2 (derived from FR-900098) were recently synthesized ^79^. They will be referred to as SP1 to SP6 in this work ( Figure S4). The goal was to enhance the bioavailability of both parent compounds by overcoming barriers, such as poor uptake of the drug by target organisms. After entering the cell by diffusion through the cell wall and transport through the PM, the drug should be released by esterases and chemical hydrolysis. In addition, the drug has to overcome a second barrier within BY-2 cells, the plastid envelope, to reach its target enzyme. The experiments were performed for two major reasons: First of all as a proof of concept to validate the bioassay with yet untested inhibitors. Second, it was desirable to see how the prodrugs will act in comparison to the reference compound Fos or the direct drug “role models”, LK1 and LK2. It was also important to see whether the bioassay may prove to be valuable for a statistical approach. As all inhibitors, with the exception of LK1, were found to be totally inhibitory at 100µM we started testing six prodrug derivatives of LK1 at 50µM ^79^ to detect a first “all-or-nothing” effect. At 50µM, all the six prodrugs were able to induce a mislocalization of the H _6_-GFP-BD-CVIL fusion protein in at least 50% of treated cells. The cells were counted by the operator of the fluorescence microscope at low magnification (using a 10x apochromatic objective) and divided into three major groups-according to their respective phenotype – to facilitate a statistical evaluation ( Figure 6a):

1. Cells in which the fluorescence emitted from the nucleus was clearly dominant;

2. Cells with significant intensity of fluorescence still emitted from the PM (nucleus and membrane were more or less equal in the distribution of fluorescence);

3. Cells in which the fluorescence was mainly associated with the PM and looking like the untreated control (the important part being that no fluorescence was visible in the nucleolus).

The results obtained at a final concentration of 50µM indicated that most of the prodrugs, especially SP2 and SP4, caused a fluorescence shift comparable to that observed after treatment with 50µM Fos ( Figure 6b). Among the six prodrugs, SP2 and SP4 appeared to be very active. In both cases, more than 80% of counted cells showed a dominant fluorescent signal from the nuclear region of the cell, indicating a significant inhibition of GGPP biosynthesis through the MEP pathway. The other compounds did not act so efficiently, such as Fos. In summary, a general tendency could be observed, clearly indicating that the methylated analogs of the respective prodrugs were far more efficient than their non-methylated counterparts. This corroborates the results obtained with LK1 and LK2 (cf. ^76^) as well as those obtained with Fos and FR-900098 (cf. ^78^).

However, at 50µM it was not possible to determine whether the prodrug SP2 was a better inhibitor than Fos. To address this issue, we therefore conducted a second set of experiments, using the inhibitors at a final concentration of 5µM. At this concentration, two major results are noteworthy ( Figure 6c): First of all, the efficiency of Fos to induce a mislocalization of the fusion protein drops dramatically. More than 30% of the cells treated with 5µM Fos did not show any fluorescence translocation to the nucleus and gave a phenotype similar to that of the untreated control cells. In addition, the percentage of cells with a dominant fluorescence signal emitted by the nucleus (among the remaining cells with a partial mislocalization) decreased by more than 50%. In contrast, SP2 as well as SP4 only showed a slightly reduced efficiency to induce a dominant mislocalization of the GFP fusion protein from the PM to the nucleus. For SP2, 72% of the cells were still significantly inhibited by the treatment at 5µM. As for SP4, the fact that over 90% of cells had mislocalization patterns indicates that there was almost no detectable loss of efficiency after a 10-fold dilution from 50 to 5µM.

## Discussion

### Treatment with inhibitors of the sterol biosynthetic pathway

The inhibition of the sterol biosynthetic pathway was used as a tool to check whether a possible re-direction of the metabolic flux in BY-2 cells, in particular FPP molecules non-incorporated into sterols, could lead to a change in the GFP fluorescence pattern or even contribute to overcome the Fos-induced mislocalization of the H _6_-GFP-BD-CVIL, reported earlier. The fact that MVA, at high concentrations, was able to rescue the fluorescence at the PM, whereas farnesol (Fol) only partially complemented the inhibition by Fos suggests (among other possibilities) the existence of a bottleneck for the synthesis of GGPP from FPP in the cytosol (no GGPP synthase expression, under our conditions), or the incapacity of FPP to be translocated into the plastid, where the formation of GGPP could take place.

This bottleneck is perhaps HMG-CoA reductase (HMGR), which is generally considered to catalyze the rate-limiting step in sterol synthesis, for both animals ^2^ and plants ^7, 8, 80^. This is supported by the fact that tobacco plants over-expressing HMGR were shown to produce higher amounts of sterol intermediates and end products ^81, 82^, whereas the over-expression of a FPP synthase in *Arabidopsis* did not result in an increased production of sterols ^83^.

Due to previous experiments with sterol biosynthesis inhibitors we had some experience on the effective concentrations of SQ and Tb in BY-2 cells ^38^. SQ was shown to be a very efficient inhibitor of SQS (IC _50_ = 5.5nM), almost completely inhibiting SQS activity after treatment for 24 h at 50nM. In addition, the same concentration of SQ led to a 97% decrease in total radioactivity incorporated into sterols, when radiolabeled [ ^14^C] sodium acetate was added for 2 h before cell harvesting. At 0.5µM, the cells showed a significant decrease in cell mass (50%), which correlates very well with the stunted growth observed during this study. In a preceding study it was demonstrated that the presence of SQ led to a growth arrest of BY-2 cells in the early G1 phase, but did not cause any cytotoxic effect or apoptosis ^26^. Inhibition of SQS by SQ was also shown to trigger a stimulation of HMGR expression and activity ^38^.

Inhibition by Tb was revealed to cause a significant decrease in the cell sterol content as well as a massive accumulation of squalene, which accumulated in cytosolic lipid bodies. However, the cell growth was not impaired as indicated by the analysis of the cell mass after treatment with 30µM Tb for 24 h. Interestingly, Tb treatment also stimulated HMGR activity, but not the corresponding transcript levels ^38^.

Keeping all this in mind, how can the particular phenotype observed with SQ-treated cells, i.e. the partial mislocalization of the GFP fusion protein be explained? In fact, in a first set of experiments, we added 20µM exogenous Fol to BY-2 cells treated with both Fos and SQ. In cells treated with SQ only (control experiment), a partial mislocalization of the fluorescence was observed in the majority of treated cells (> 85%), whereas neither Tb nor Ro-treated cells displayed a similar effect. In cells treated with both SQ and Fos and complemented with Fol, the same pattern of partial mislocalization of the fluorescence was observed. It needs to be pointed out that this mislocalization was clearly visible, but less pronounced at the level of a single cell, as compared to Fos- or OC-treatments.

Hypothetically, due to SQS inhibition, the redirection of the metabolic flux might provide much more FPP to other FPP-metabolizing enzymes, such as GGPP synthase or protein farnesyltransferase. FPP in excess could also be transported to mitochondria (cf. ^84^) or other organelles or converted to Fol, which might become cytotoxic ^85, 86^. Feeding experiments with radiolabeled farnesol showed that all these scenarios could occur. However, inhibition with SQ did not lead to an additional incorporation of FPP into the ubiquinone side-chain ^84^.

Thus, a protein farnesyltransferase (PFT) might use the excess FPP to non-specifically farnesylate the H _6_-GFP-BD-CVIlL fusion protein, despite its geranylgeranylation motif. Some *in vitro* cross-reactivity of PFT from *Arabidopsis* has been suggested ^87^, however such observations might not reflect the situation *in vivo*. By the same token, a PGGT-1 might accept FPP as substrate if present at concentrations that are much higher than that of GGPP. When the CVIL motif in our fusion protein was mutated to CVIM, thus transforming it into a potential substrate for PFT, it resulted in a fluorescence pattern with the majority of fluorescence still being associated to the PM of the cell, but also significantly present in and around the nuclear membrane, with less in the nucleus and nucleolus ^88, 89^. As the basic domain of the chimeric protein not only serves as a second signal for protein prenylation (besides the CaaX motif), but also contains a putative nuclear localization sequence (NLS), it is possible to imagine that the farnesyl moiety is not sufficient either to guarantee an efficient integration in the PM (because of a shorter hydrophobic domain) or to mediate the transport to the PM (which is still an open question for H _6_-GFP-BD-CVIL). In addition, the occurrence of several farnesylated, nuclear proteins have been recently reported ^90^. Very recently Chandra *et al.* ^91^ identified a role for the GDI-like solubilizing factor (GSF) photoreceptor cGMP phosphodiesterase δ subunit δ(PDEδ) in modulating signaling through Ras family G proteins by sustaining their dynamic distribution in cellular membranes. In this study it was shown that the GDI-like pocket of PDEδ binds and solubilizes farnesylated Ras proteins, thereby enhancing their diffusion in the cytoplasm ^91^. This would allow more effective trapping of (depalmitoylated) Ras proteins at the Golgi and polycationic Ras proteins at the plasma membrane ^91^.

Studies with SQ in mammalian systems revealed that besides acting as a specific inhibitor of SQS, SQ was able to inhibit mammalian prenyltransferases, in particular protein farnesyl transferase (PFT) ^39, 92^. PFT and PGGT-1, purified from bovine brain, were inhibited by SQ *in vitro* with an IC _50_ of 216 and 620nM, respectively ^92^. As we applied comparably high concentrations of SQ (500nM to 1µM) to BY-2 cells, it seems quite reasonable to assume that the treatment with SQ might also have affected the PGGT-1 enzyme *in vivo*, responsible for the prenylation of the H _6_-GFP-BD-CVIL, even though *in vitro* data are not yet available. In addition, by using lower concentrations of SQ (50nM already blocked most of SQS activity), the protein prenyltransferase should theoretically not be inhibited, allowing us to more or less rule out the cross-reactivity and the farnesylation scenario. In order to investigate the capability of isoprenols like geraniol (Gol), farnesol (Fol) and geranylgeraniol (GGol) and intermediates of both isoprenoid biosynthetic pathways (MVA-pathway: mevalonolactone (MVL) and its open-acid form MVA; MEP-pathway: DX) to overcome the (partial) mislocalization induced by SQ, different chemical complementation experiments were performed. Interestingly, both DX and GGol completely complemented the SQ-induced effect. As discussed extensively before, the most likely explanation is an efficient uptake and metabolization of both compounds: DX, after being converted to DXP by cytosolic xylulose kinase ^30^ enters the plastid and is incorporated into IPP, GPP and GGPP, which might then be exported towards the cytosol, where it serves as a substrate for prenylation of the H _6_-GFP-BD-CVIL. For GGol, again, two scenarios are possible: either its import into the plastid, phosphorylation and export to the cytosol, or direct phosphorylation in the cytosol. The existence of a system for phosphorylation of prenyldiphosphates was mentioned previously ^93^.

SQ is known to be an irreversible, competitive inhibitor of squalene synthase (SQS). Therefore, the DX-induced recovery of the PM fluorescence itself cannot result from an effect on SQS. As SQ structurally resembles FPP, it would be more likely that, by increasing the pool of bio-available GGPP, we succeeded in replacing SQ by GGPP at the substrate-binding site of PGGT-1 (or even both prenyltransferases) and thereby restored the correct geranylgeranylation of H _6_-GFP-BD-CVIL.

Interestingly, mevalonate kinase is down-regulated at the post-transcriptional level by high levels of FPP and GGPP ^94^. Therefore, if the tobacco BY-2 mevalonate-kinase were also sensitive to the feedback-regulation by FPP and GGPP, this would very conveniently explain why externally fed MVA could not complement the SQ-induced phenotype due to a large pool of FPP. Moreover, the resulting decrease in cytosolic IPP due to the block in the MVA pathway could also prevent a sufficient supply of IPP for the putatively cytosolic conversion of FPP to GGPP.

The fact that Gol does not complement the effects of SQ is in agreement with previous results ^88^ and suggests that Gol is not accepted as a substrate for the phosphorylation system that exists in plants ^93^. Intriguingly, exogenously applied Fol was previously able to restore the PM localization of H _6_-GFP-BD-CVIL to over 60% in Fos-treated cells ^88^. However, as there are very specific inhibitors that act either on PFT or on PGGT-1, and are thus useful for “calibration”, in a follow-up experiment an unknown inhibitor giving a “hit”, but then applied at much lower concentration and carrying out similar complementation assays, would easily distinguish between inhibition of sterol biosynthesis or protein prenylation.

### Transport of prenylated H _6_-GFP-BD-CVIL – post-prenylation effects?

Some previous studies in our laboratory focused on the intracellular transport of the prenylated GFP-fusion protein from the ER to the PM in BY-2 cells ^88^. However, the transport of GFP-BD-CVIL was not significantly affected by any treatment targeting either components of the cytoskeleton (microtubules: taxol and oryzalin; actin-filaments: cytochalasin D) or the *trans*-Golgi-network (TGN: brefeldin A ^88^), suggesting that GFP-BD-CVIL was not transported to the PM *via* the classical secretory pathway. This result was surprising, as this vesicular transport route is used by the majority of identified prenylated proteins ^95^, such as HRAS, NRAS and KRAS4A in mammals. But the correct targeting of Ras proteins is successfully accomplished only when the proteins contain a second “signal” in addition to their prenylation. In the case of HRAS, NRAS and KRAS4A this second signal consists of one or more *S*-acylation sites (“palmitoylation”) ^96, 97^, whereas KRAS4B contains a lysine-rich polybasic (hypervariable) domain, located nearby the prenylation side ^98– 100^. The same combination, a polybasic domain with a cluster of basic amino acids, in addition to the CAAX motif is also found in the H _6_-GFP-BD-CVIL fusion protein.

Besides this structural resemblance, KRAS4B is also insensitive to inhibitors of vesicular transport and transits to the PM by a largely unknown, non-vesicular (Golgi-independent) pathway ^95, 101, 102^ that requires, at least in yeast, mitochondrial function and vacuolar sorting proteins (VPS) ^103^.

Despite the fact that corresponding experimental efforts with transformed BY-2 cells were not crowned by much success ^88^, the study of molecules that could interfere with the transport of the H _6_-GFP-BD-CVIL fusion protein might be a promising tool to observe the effects of post-prenylation inhibition in plants in general, and this knowledge might even be transferred to other models.

### Application of the BY-2 cell system for the test of synthetic inhibitors of MEP pathway enzymes

Overall, two sets of experiments have been conducted in the context of validating the test system. Two groups of novel inhibitor candidates, each targeting a specific enzyme of the MEP pathway, DXS in the first and DXR in the second case, were tested to assess their capacity to inhibit the *in vivo* isoprenylation (more precisely: geranylgeranylation) of the chimeric H _6_-GFP-DB-CVIL protein.

First, six compounds that were inspired by known inhibitors of pyruvate decarboxylases and pyruvate dehydrogenases were tested. In summary, all tested pyruvate derivatives were inefficient in our *in vivo* assay, despite the fact that one of them, TJ150, induced cytotoxic effects at extreme concentrations ( Figure 4). At this point two scenarios were plausible: either their uptake into the cells and subsequent transport through the plastidial envelope might have been impaired or they did not inhibit their molecular target, DXS. However, in parallel to the tests conducted with the H _6_-GFP- BD-CVIL cell line, *in vitro* DXS activity from *E. coli* was determined in the presence of some of the tested molecules using an end-point assay, in which the remaining pyruvate substrate was converted to lactate by lactate dehydrogenase. The consumption of NADH (and the formation of NAD ^+^) can be measured by spectrometry at 340nm (cf. ^57^). None of the tested compounds exhibited an inhibitory effect, in agreement with the results obtained with our bioassay (D. Tritsch, unpublished observations).

The second set of experiments focused on inhibitors and novel prodrugs targeting the second enzyme of the plastidial pathway, DXR. Only recently, analogs of Fos and FR-900098 with a rearrangement of the hydroxamate group had been tested *in vitro* ^76^. Those compounds (here referred to as LK1 and LK2) successfully inhibited *E. coli* DXR. The IC _50_ values measured were 170nM and 48nM for LK1 and LK2, respectively. The IC _50_ value of LK2 was even comparable to that of Fos (32nM). In addition, both molecules inhibited the growth of *E. coli*, but with a lower efficiency than Fos ^76^. Interestingly, the N-methylated compound (LK2 – derived from FR-900098) also successfully inhibited the growth of an *E. coli* strain that was cross-resistant to Fos and fosfomycin, which bears some faint structural similarity, but acts on another molecular target, UDP-N-acetylglucosamine enolpyruvyltransferase ^104^. Among other reasons, this could be due to a difference in the uptake or detoxification of these two antibiotics, which are known to share a common import mechanism via the L-3-glycerolphosphate and the glucose 6-phosphate pathway in *E. coli* ^105^. As the N-methylated compound LK2 nearly equaled Fos in terms of *in vitro* inhibition of the DXR enzyme ^76^, changing the bioavailibility of this compound was a promising strategy to further enhance the performance of the hydroxamic drugs and provide new active compounds against resistant pathogen strains.

**Figure 7.**
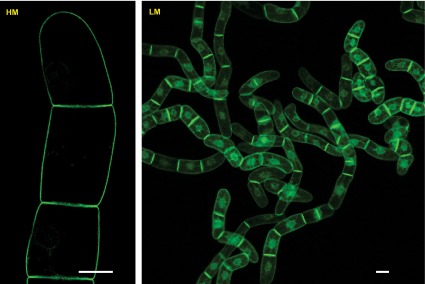
Clonal transgenic tobacco BY-2 cell line expressing the H _6_-GFP-BD-CVIL fusion protein. GFP expression was induced according to the previously described standard protocol. Both images were taken with a confocal laser scanning microscope (Zeiss LSM510 equipped with an inverted Axiovert 100M). HM-High magnification image taken with a 63x water immersion objective (C-Apochromat 63x/1.2W M27), which is specifically designed for the examination of an aqueous specimen and delivers high resolution in optical thin sections, with satisfying levels of fluorescence brightness. LM-Low magnification image taken with a 10x "Enhanced Contrast Plan-Neofluar" universal objective (EC Plan-Neofluar 10x/0.3 M27), adapted for general observations in fluorescence microscopy. If not otherwise stated, the label HM and LM designates the use of these both types of objectives for the image acquisition with the confocal laser scanning microscope. White bars = 20µm. Please, note the different saturation of the images and the significantly higher saturation in the LM image that was used to highlight the homogenous levels of bright fluorescence of the re-selected clonal cell line.

The *in vivo* experiments with transformed BY-2 cells were conducted under similar conditions: clonal cells tested under pre-defined standard conditions were counted and examined by visual inspection after different treatments (cf. Figure 7).

The test of the six new prodrugs ^79^, here referred to as SP1 to SP6, includes a whole new aspect: for the first time, novel drugs specifically designed on the basis of known pathway inhibitors were tested under standardized conditions and the generated data were exploited in both a qualitative and a quantitative/statistical way. This means that, besides answering the question of whether an inhibitor candidate could efficiently block the MEP pathway, the exploitation of the same set of data would allow a direct comparison of the efficiency of this compound with established MEP pathway inhibitors. By testing the inhibitors at different concentrations, we were able to first define a starting point for the experiments, which consisted in a concentration that inhibited the majority of cells (> 95% of cells showing a dominant mislocalization). The results obtained at 100µM clearly proved that Fos and FR-900098 as well as LK2 were applied at saturating concentrations. We therefore decided to better start our direct comparison at 50µM. At this concentration, Fos, SP2 and SP4 were very active, causing a partial mislocalization in nearly 100% of the cells. The other tested prodrugs did not act that well at this concentration, even though they were able to induce a partial mislocalization in at least 50% of treated cells. At 5µM, only SP2 and SP4 proved to be more efficient than Fos, whereas the other inhibitors only induced a barely detectable effect. At 500nM final concentration, SP2 and SP4 were still able to induce a mislocalization of the GFP fluorescence in more than 50% of the cells (data from a single experiment).

**Figure S1.**
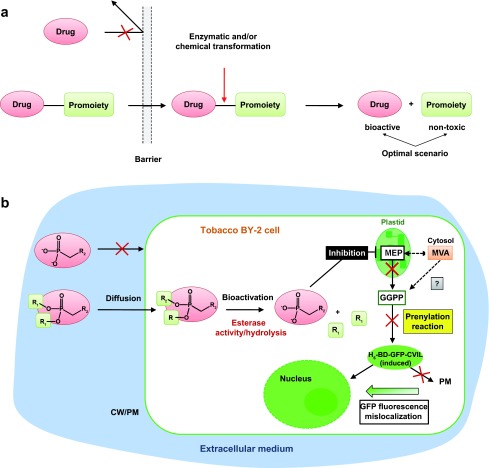
Simplified illustration of the prodrug concept in context with the fluorescent reporter system based on the prenylable and inducible GFP fusion protein H _6_-BD-GFP-CVIL (modified from Rautio *et al.* ^107^). The prodrug is a chemically modified version of the pharmacological active compound. The addition of a covalently linked “promoiety” masks the parent drug and improves for example its physicochemical or pharmacokinetic properties. **A**) If the parent drug has difficulties overcoming a barrier, such as a biological membrane or is poorly soluble, the addition of a promoiety can be seen as a strategy to overcome this barrier. In an optimal scenario, the prodrug releases the active drug after removal of the promoiety by enzymatic or chemical transformation (“bioactivation”). The drug should ideally be recovered at high rates and the promoiety should be non-toxic. **B**) In the case of the inhibitors synthesized and described elsewhere ^79^ in the model system BY-2, the prodrugs are ester derivatives of phosphonohydroxamic drugs. After entering the cell by diffusion through the cell wall (CW) and transport through the plasma membrane (PM), the drug is supposed to be released by esterases and/or chemical hydrolysis. In addition, the drug has to overcome a second barrier within the cell, the double membrane of the plastid, to reach its target within the plant cell. Inhibition of DXR activity leads to a dramatic change in the pool of relevant precursors for the geranylgeranylation of H _6_-BD-GFP-CVIL. This terpenoid precursor, GGPP, is almost completely derived from the plastidial MEP pathway in our test system. Without the prenylation of the C-terminal moiety, the fusion protein is likely to mislocalize from the PM (and to some extent the nuclear membrane) to the nuclear compartment, which is due to the presence of a nuclear localization sequence (NLS) in the truncated peptide derived from OsCaM61 (for further explanation: Gerber *et al.* ^1^).

Obtaining only two out of the six compounds with a higher efficiency than the respective model drugs might appear deceiving at first view. However, it is a fine example confirming the prodrug concept ( Figure S1) ^106, 107^. The efficiency of a prodrug in an *in vivo* assay indeed depends on several parameters, such as transfer through biological membranes and the stability of the prodrug against hydrolysis or esterases.

All the six prodrugs tested in these experiments were able to induce a localization shift of the prenylable GFP fusion protein to some extent, which indicates that the active drug at least partially entered the plant cell, where it could inhibit the DXR enzyme (located in the plastidial compartment). This point is important to mention, as it indicates that:

i) The prodrugs were soluble in the BY-2 medium;

ii) They successfully crossed the plant cell wall;

iii) They were imported inside the plant cell across the PM.

How this transport took place and which transporters were involved remains unclear, even though data from bacteria suggest that a cAMP-dependent glycerol 3-phosphate transporter could be a likely candidate ^108^. Until now, a similar transporter has only been identified in the mitochondrial membrane of plant cells ^109^.

**Figure S2.**
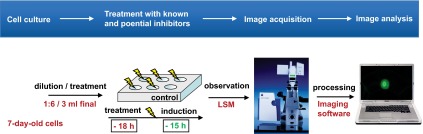
Setup for the bioassay developed during preceding studies ^88^ and used as basis for the experiments in the present work. The test system is based on a stably transformed tobacco BY-2 cell line expressing the chimeric, prenylable fusion protein H _6_-BD-GFP-CVIL. The expression is under the control of a dexamethasone-inducible promoter ^116^. The usual timeline for an experiment consisted of 3 h treatment followed by an induction of protein expression varying between 15–48 h depending on the experimental setup. During that period the cells were grown under constant shaking in the dark at 27°C before the phenotype was analyzed using confocal laser microscopy.

By relating the results to the structural features of the tested molecules, another important observation can be made. The data obtained for all inhibitors showed that the methylated prodrugs displayed a far better activity than their non-methylated counterparts. This result correlates with observations obtained with Fos, FR-90098, LK1 and LK2 in bacterial systems ^76, 110^. Interestingly, the best performing methylated prodrug SP4 also shared the same pro-moieties with the best performing non-methylated prodrug SP3. Although the better performance of the methylated drugs, such FR-900098 or LK2, could be attributed to the hydrophobic interaction of the methyl-group at the catalytic site of the DXR enzyme, the methyl group may also have an influence on the lipophilicity of the prodrug and be responsible for its overall better import rate in tobacco BY-2 cells ( Figure S2). Other factors could be the different preferences of endogenous esterases for the three types of substituents used to synthesize the prodrugs or the rate of spontaneous hydrolysis (that may occur either in the extracellular medium or the cytosol).

It should be noted that the best performing prodrugs in the bioassay with BY-2 cells showed the same order of efficiency when tested on *Mycobacterium smegmatis* cells, suggesting that knowledge gained from plant cells might be transferred to other organisms ^79^. Nevertheless, keeping in mind the potential of MEP pathway inhibitors as herbicides, these results are very promising and set the course for the development of a chemical drug screen, with both biomedical and agricultural applications, particularly in the light of newly emerging strains of multi-drug resistant bacterial pathogens.

**Figure S3.**
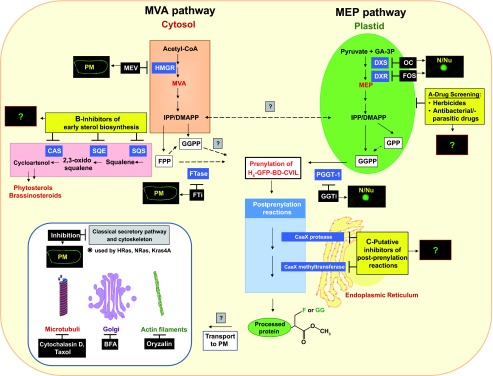
Overview of the treatments in the context of previous results obtained with a test system based on the fluorescent reporter protein H _6_-GFP-BD-CVIL in transgenic tobacco BY-2 cells. In previous studies, the following phenotypes were observed after treatments of the transgenic H _6_-GFP-DB-CVIL cell line with inhibitors of both isoprenoid biosynthetic pathways: Inhibition of the cytosolic MVA pathway by mevinolin (MEV) a specific inhibitor of HMG-CoA reductase (HMGR) did not exhibit any detectable effect on the intracellular localization of the prenylable GFP fusion protein which predominantly associated with the plasma membrane (PM), as observed under control conditions. Specific inhibition of DXS and DXR, key enzymes in the plastidial MEP pathway by oxoclomazone (OC) and fosmidomycin (Fos), respectively, however lead to a dramatic mislocalization of the GFP fluorescence (compare main text for more detailed information) to the nuclear compartment (N/Nu). A similar effect was observed with compounds that interfere with protein geranylgeranyltransferase type I (PGGT-1). This differential localization of the GFP fusion protein in response to inhibitors of the two coexisting routes leading to the synthesis of the universal C5 precursors IPP (and its isomer DMAPP) in plants indicates the suitability of this system for screening of compounds with potential antimicrobial and herbicidal activities. In the present study this potential for drug screening will be addressed by testing novel compounds, specifically designed to inhibit DXS and DXR enzyme activity, thereby validating the test system (yellow box A). In addition, the impact of various inhibitors targeting enzymes involved in the early steps of phytosterol biosynthesis, one of the main routes of cytosolic FPP within the plant cell, will be investigated (yellow box B). Finally, the effect of post-prenylation inhibitors on the localization/transport of H6-GFP-DB-CVIL is addressed, especially in the light of prior experiments aimed at clarifying the details of its yet unknown transport to the PM (yellow box C). These experiments have to be seen in the context of the fate and intracellular transport of typical mammalian isoprenylated proteins, such as members of the RAS family, that play an important role in the development of certain varieties of cancer (compare with discussion in the main text).

**Figure S4.**
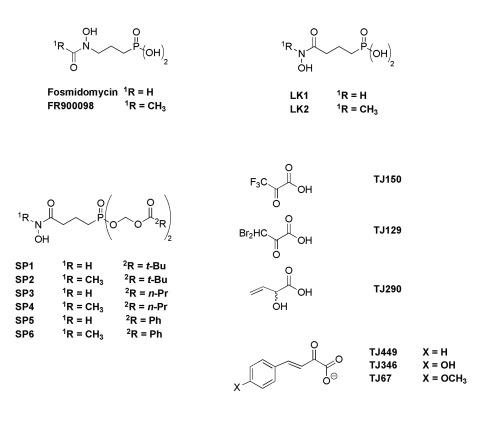
Structures of newly synthesized compounds and tested in this study.

The experimental approaches used and applied to the specific situation in tobacco BY-2 cells synthesizing isoprenoids *via* two compartmentalized pathways and expressing the reporter GFP fusion protein are summarized in Figure S3. The scheme indicates also some more hypothetical links to observations in mammalian cells, for instance as to the post-prenylation reactions and the transport of covalently modified proteins to their intracellular destination.

## Methods and materials

### Chemicals

If not indicated otherwise, chemicals used were provided by Sigma-Aldrich/Fluka. Squalestatin (Sq) was obtained from Glaxo (Greenford, Middelsex, UK) and dissolved in 0.1M Tris-HCl (pH 7.4) to give a 0.1mM stock solution. Terbinafine (Tb) was kindly provided by Dr. N.S. Ryder (Vienna) and dissolved in dimethyl sulfoxide. MVA lactone, Gol, Fol, and GGol were purchased from Sigma-Aldrich. Mevinolin (MV) was a kind gift from Dr. Alfred Alberts (MSD, Rahway, NJ). Before use, the lactones of MV and MVA were converted to their open acid forms. Fosmidomycin (Fos) ^111^ was made available by Robert J. Eilers (Monsanto, St. Louis, MO). The peptidomimetic protein geranylgeranylation inhibitor GGti-2133 ^112^ was purchased from Calbiochem (Merck). Oxoclomazone (OC) was kindly supplied by Dr. Klaus Grossmann (BASF, Limburgerhof, Germany). Please see Table 1 for a list of compounds and their final concentration used in this study

**Table 1.** List of inhibitors and other compounds used in this study.

Prodrugs – DXR (Abbreviation)	*M*_r_	Solvent	Stock solution	Final concentration
**SP1**	411.38	MeOH	100mM	0.5–50µM
**SP2**	425.41	MeOH	100mM	0.5–50µM
**SP3**	383.33	MeOH	100mM	0.5–50µM
**SP4**	397.36	MeOH	100mM	0.5–50µM
**SP5**	451.35	MeOH	100mM	0.5–50µM
**SP6**	465.39	MeOH	100mM	0.5–50µM
Potential inhibitors – DXS (Abbreviation)	*M*_r_	Solvent	Stock solution	Final concentration
**TJ449**	176.17	H _2_O	100mM	100µM
**TJ129**	142.03	H _2_O	100mM	100µM
**TJ346**	192.17	CH _3_CN	100mM	100µM
**TJ67**	206.19	CH _3_CN	100mM	100µM
**TJ150**	245.85	H _2_O	100mM	100µM
**TJ290**	102.09	H _2_O	100mM	100µM
Other inhibitors (Abbreviation)	*M*_r_	Solvent	Stock solution	Final concentration
**Fosmidomycin (FOS)**	183.10	DMSO	100mM	5–100µM
**FR-900098**	197.13	DMSO	100mM	up to 100µM
**LK1**	183.10	H _2_O	100mM	up to 100µM
**LK2**	197.13	H _2_O	100mM	up to 100µM
Farnesyl thiosalycilic acid (FTS)*	358.5	DMSO	28mM	2µM
N-Acetyl-S-geranylgeranyl- L-cysteine (AGGC)*	435.7	EtOH	23mM	2µM
S-(5'-Adenosyl)- L-homocysteine (AHC)*	384.4	H _2_O	2.6mM	2µM and 20µM
**N-Acetyl-S-farnesyl-L-cysteine (AFC)**	384.58	MeOH	50mM	20–200µM
**N-Tosyl- L-phenylalanine chloromethyl ketone**	351.8	DMSO	20mM	20µM
**Squalestatin**	690.73	KH _2_PO _4_(50mM)	1mM	1µM
**Ro48-8071**	448.40	methyl acetate	2mg/L	2µg/ml
**Terbinafine**	291.43	DMSO	27.5mM or 8mg/ml	up to 30µM
Complementation Treatments (Abbreviation)	*M*_r_	Solvent	Stock solution	Final concentration
**1-Deoxy- D-xylulose**	134.133	H _2_O	150mM	0.5mM
**Mevalonolactone (MVL)**	130.14	H _2_O	1.15M	5mM
**Mevalonic acid (MVA)**	148.16	see text	3M	3mM
**Geraniol (Gol)**	154.25	EtOH	10mM	5 and 20µM
**Farnesol (Fol)**	222.37	EtOH	20mM	5 and 20µM
**Geranylgeraniol (GGol)**	290.48	EtOH	20mM	5, 10 and 20µM
**Squalene**	410.72	stock solution	~2M at 25°C	~2 and 4mM

*AHC, AGGC and FTS were also tested in initial screening experiments but showed no obvious effects on the localization of the GFP fluorescence under the chosen experimental conditions. FTS treatment however resulted in a fewer amount of fluorescent cells.

### Plant materials: Tobacco BY-2 cells

The original untransformed tobacco ( *Nicotiana tabacum* L.) cv Bright Yellow-2 (BY-2) cell line used in this work was provided by Professor Toshiyuki Nagata (University of Tokyo, Japan). It was initially isolated by Kato *et al.* ^113^ and is derived from calli induced from young plants ^114^. The cell suspension was maintained by subculturing the 7-day-old cells (in stationary phase) as described ^1, 28, 85^.

The transgenic dexamethasone-inducible Tobacco BY-2 H _6_-GFP-BD-CVIL cell line has been extensively described by Gerber *et al.* ^1^ and was maintained as described.

Routinely, 0.75ml of a 7-day-old culture was diluted into 40ml of modified Murashige and Skoog medium ^115^. The cells were then cultured at 27°C in the dark on a rotary shaker (154 rpm).

### Inducible expression of GFP fusion protein and treatments

The inducible test system (cf. ^116^) has been adjusted to the 7-day growth cycle of TBY-2 cells. After 6-fold dilution in fresh medium, the cells were treated in commercial multi-well plates. Total volume of treated cells was scaled to 3ml. In general, initial screenings started between 50 and 100µM of a given inhibitor/prodrug and were refined if a delocalization occurred. Induction of the fluorescent marker proteins took place 15 to 24 h before observation; the treatments were scheduled 3 to 6 h after the induction. Observation and image acquisition were made by laser scanning microscopy as described ( Figure S2).

### Microscopy and cell imaging

Cell imaging was performed using a LSM510 confocal laser scanning microscope equipped with an inverted Zeiss axiovert 100M microscope (Carl Zeiss, Jena, Germany). For statistical observations, such as counting cells after various treatments, a 10x Zeiss objective (“Plan-Neofluar”) was used. At confocal resolution, images were taken using a 63x, 1.2 numerical aperture water immersion objective (“C-Apochromat”). In both cases, differential interference contrast (DIC) images were recorded.

Epifluorescence microscopy was performed using a Nikon E 800 fluorescence microscope equipped with a Nikon DXM1200 CCD high-resolution color camera (20 x 0.45 objective) and ACT-1 image software. Images were processed with the latest version of the Zeiss LSM Image Browser software (Carl Zeiss, Jena, Germany) and exported as Tiff or JPEG files. Final image analysis and adjustments was performed using either Photoshop version 8.0 (Adobe Systems, San Jose, USA) or the ImageJ software version 1.31 (Wayne Rasband, NIH, USA).
