# Supplementary material for: The effect of MEP pathway and other inhibitors on the intracellular localization of a plasma membrane-targeted, isoprenylable GFP reporter protein in tobacco BY-2 cells
Source: F1000Res. 2013 Nov 15;2:170. Originally published 2013 Aug 12. [Version 2] doi: 10.12688/f1000research.2-170.v2 (PMC3886798; doi:10.12688/f1000research.2-170.v2)
Supplement: Peer review history

# Peer review: Referee response for version 2

**Published:** 2013-11-20

**Authors:** Angelos Kanellis

**Affiliations:** 1 Department of Pharmaceutical Sciences, Aristotle University of Thessanoliki, Thessaloniki, Greece

I believe the authors have successfully addressed the reviewer comments and thus, I again approve the publication of this article.

I have read this submission. I believe that I have an appropriate level of expertise to confirm that it is of an acceptable scientific standard.

# Peer review: Referee response for version 2

**Published:** 2013-11-19

**Authors:** Ivo Feussner

**Affiliations:** 1 Department of Plant Biochemistry, Georg-August-University, Göttingen, Germany

The authors have answered my questions and I have no further comments.

I have read this submission. I believe that I have an appropriate level of expertise to confirm that it is of an acceptable scientific standard.

# Peer review: Referee response for version 1

**Published:** 2013-11-06

**Authors:** Angelos Kanellis

**Affiliations:** 1 Department of Pharmaceutical Sciences, Aristotle University of Thessanoliki, Thessaloniki, Greece

The article entitled “ *The effect of MEP pathway and other inhibitors on the intracellular localization of a plasma membrane-targeted, isoprenylable GFP reporter protein in tobacco BY-2 cells*” by Hartmann *et al*., describes the use of an in vivo system to study the effectiveness of known inhibitors of MEP and MVA pathways and also to screen for new chemicals that affect these pathways with the hope of eventually being able to use them as herbicides, antibacterial/ant-parasitic drugs and as inhibitors of early sterol biosynthesis and of post-prenylation reactions.

 

The experiments were well designed and executed and showed that this is a promising system that can be used not only for the screening of new inhibitors of the MEP pathway, but also to study the possible channelling of the intermediates to different compartments and/or to follow the interplay between the two pathways. The latter can also help with applying metabolic engineering to terpenoid production in plants or microorganisms.

I have read this submission. I believe that I have an appropriate level of expertise to confirm that it is of an acceptable scientific standard.

# Response

**Published:** 2013-11-13

**Authors:** Thomas J. Bach

**Affiliations:** IBMP, CNRS UPR 2357, Université de Strasbourg, France

We are quite grateful for the very positive comments by this colleague, who has provided a broader and deeper perspective to our article than might at first be obvious by a superficial read of the manuscript.  It is clear that he is a specialist in the realm of plant isoprenoid research.

# Peer review: Referee response for version 1

**Published:** 2013-09-04

**Authors:** Michael Pirrung

**Affiliations:** 1 Department of Chemistry, University of California, Riverside, CA, USA

This is an interesting chemical genetics study addressing the parallel biosynthetic pathways for isoprenoids in plants (which are also significant in human pathogens). As such, this work provides a paradigm for the discovery of bioactives that might be applied in several contexts. Of course, the unique characteristics of plant cell walls / membranes, contrasted with bacteria, may make this screen less general than might be desirable. Being based on imaging, this screen is relatively low throughout, but cell-based assays typically have some of this sort of limitation. A few new chemotypes have been investigated and found to have significant activity. This study follows well-established processes for examining the sequence of steps in a biosynthetic pathway, with opposing effects of inhibitors and molecules downstream of the site of inhibition. 

A slight ambiguity about the chemical structures of the pro-drugs and their active forms hinders understanding of the structure-activity relationships. It should be emphasized that the most active SP compounds have N-methyl groups on their hydroxamate nitrogens. Also, structures presented on p. 20 are in error, in that all but TJ290 are missing a carbonyl group.

I have read this submission. I believe that I have an appropriate level of expertise to confirm that it is of an acceptable scientific standard.

# Response

**Published:** 2013-11-13

**Authors:** Thomas J. Bach

**Affiliations:** IBMP, CNRS UPR 2357, Université de Strasbourg, France

The referee went quite deeply into the details of the manuscript and we are very grateful for his sharp eyes, detecting a mistake in the supplementary figure summarizing some (synthetic) inhibitors checked in our study. The corresponding figure will of course be replaced.

 

The colleague points to some limits of such a system, but we have prepared a sequel manuscript also to be sent to F1000research, in which all has been “miniaturized” to the use of 96-well plates and a semi-automatic analysis by confocal microscopy. It was necessary however, to shown that the reporter system as such, based on this GFP fusion protein works sufficiently well before publishing the second manuscript. As to the efficacy of compounds that interact with the MEP pathway or with downstream reactions of protein isoprenylation the first criteria is to penetrate into the BY-2 cells, and *ad extremum* to get into the plastids. It is obvious and also a major result that “pro-drugs” with increased lipophilicity enter more easily, like is known for a large number of pesticides and pharmaceuticals. It is somewhat ambiguous to go too deeply into the field of structure-activity relationships, but Dr. Pirrung is right in ascribing the highest activity to a certain group of compounds, like those that have N-methyl groups and their hydroxamate nitrogens.

 

There is no “ideal” system for testing inhibitors, and this concerns those whose research is based around bacteria, as well.  Let us take for instance the well-known fosmidomycin that penetrates into *E. coli*, but not into the humano-pathogenic *Mycobacterium*. Once an inhibitor has been found efficient in our plant cell-based test system, it is clear that in follow-up experiments one has to investigate whether it acts similarly upon a whole collection of such bacteria. Human pathogens are not even limited to bacteria. Simply pragmatically speaking, a plant cell is somewhat representative of different pathogenic cells: protistans, bacteria and fungi.

# Peer review: Referee response for version 1

**Published:** 2013-08-15

**Authors:** Ivo Feussner

**Affiliations:** 1 Department of Plant Biochemistry, Georg-August-University, Göttingen, Germany

This manuscript by Bach, Rohmer and colleagues describes a new and powerful plant screening system for chemical inhibitors of the MEP or Rohmer pathway. The basic concept as well as the underlying chemistry of the manuscript is excellent, but the presentation of the results and conclusions driven from the biological experiments, as well as a number of paragraphs need carefull revision. 

A few points that shall help the authors to improve the manuscript are:

- None of the inhibitors the authors measure show their uptake. This should be mentioned/discussed.

- The size of the cells should be shown with exactly the same magnification in all figures. Otherwise it is hard to compare the morphology of the cells upon treatment with different inhibitors. To my mind it seems that the treatment does result in changes in cell size and shape i.e. Figure 1, unlike what is stated for Ro in the 7th paragraph of page 4.  In Figure 2 only the cells in the top line show stunting; the cells shown in the lines below look normal to me.

- The authors should carefully check again whether parts/paragraphs can be moved into the method section and vice versa.

- In Figure 6 fluorescence intensity is given. How was this measured, do the authors not mean distribution?

I have read this submission. I believe that I have an appropriate level of expertise to confirm that it is of an acceptable scientific standard, however I have significant reservations, as outlined above.

# Response

**Published:** 2013-11-09

**Authors:** Thomas J. Bach

**Affiliations:** IBMP, CNRS UPR 2357, Université de Strasbourg, France

This was the first comment to be delivered (thank you), but we waited until now, so as to respond to all reviews at the same time.

 

The criticism on the organization of the paper is certainly justified, but in fact it is the result of frequent exchanges of e-mails and versions with the editorial office of F1000. The initial version was a long manuscript with a very large supplement.  Upon the advice received, everything was restructured and finally two separate manuscripts were prepared, and to tell you the truth, what was a major part of the so-called supplement is now this first manuscript that describes the effects of a series of MEP pathway inhibitors and some that putatively interfere with protein isoprenylation and processing of correspondingly modified proteins.  The follow-up manuscript will provide information on the establishment of a medium-throughput system and what measures need to be taken to miniaturize the system and to facilitate microscopic analysis of rather large numbers of samples, based on BY-2 cells grown and treated in small volumes (96-well plates). We think that by then all questions that remain open in this paper will become clear, including when it comes to statistics and so forth.

 

Only if a compound is absorbed, meaning that it had passed the thin cell wall and the plasma membrane of BY-2 cells it can exert some effect in the cytoplasm (i.e., protein geranylgeranylation), or beyond after having surmounted the barrier of the double membrane that makes up the plastidial envelope to target the MEP pathway. Thus specific uptake studies, for instance with radiolabeled compounds would go far beyond the scope of this paper, not to speak of costs!

 

Dr. Feussner is right in pointing to the different size of cells as they appear in the figures. But for that reason the (high resolution) photographs made with the confocal microscope are automatically completed with white bars for scaling. We know from earlier studies with BY-2 cells treated with sterol biosynthesis inhibitors that cells become more stunted, but in this series of experiments here we searched for putative effects on the insertion of the isoprenylated GFP reporter protein and found none. (Just as an aside, in a completely different study, sterols were depleted from the membrane by cyclodextrin treatment beyond 30%, without noticeable effect on the viability of cells.)  Treatment of BY-2 cells with squalestatin (inhibitor of squalene synthase) and by terbinafin (blocks squalene epoxidase) led to similar morphological adaptations (reference #38): After cell division sterols are needed for cell elongation. However, a clear effect on the GFP reporter protein was seen with squalestatin, but this could be attributed to its direct inhibition of protein isoprenylation, as already described in literature.

 

Indeed, the treatment with Ro at the concentrations applied did not really affect the cell shape (Fig. 1a) and the statement criticized in the main text needs to be weakened.

Dr. Feussner is right: In Fig. 6 we mean fluorescence distribution, not intensity. This will be changed accordingly.

 

In Fig. 2 chemical complementation assays are displayed, with 1 µM squalestatin as the inhibitor.  (This is already a pretty high concentration of this very efficient ± irreversible inhibitor, but which also needs to penetrate into the cells.)  The only compound that really counteracted the apparent side effect on protein geranylgeranylation besides DX was geranylgeraniol (GGol). However, in previous studies we had already demonstrated that under certain conditions, when the cytoplasmic MVA pathway is blocked, in the BY-2 cell system exogenous DX can override the inhibition of sterol biosynthesis, which would result in less stunted cells. GGol, at the concentrations tested might prevent the binding of squalestatin to protein geranylgeranyl transferase. This does not hold true for MVA (open acid or lactone form), which would reconstitute sterol biosynthesis to some extent resulting in some cell elongation, but not entirely the inhibition of PGGT type I. The slight effect of geraniol might be due to its conversion into farnesyl-PP, but that is more speculation.  We think we can leave this as it is described and discussed now.
